# Analysis of Emergency Department Visits by Patients With Giant Cell Arteritis: A National Population-Based Study

**DOI:** 10.7759/cureus.35121

**Published:** 2023-02-17

**Authors:** Christopher Hino, Ehizogie Edigin, Osaigbokan Aihie, Precious Eseaton, Victory Okpujie, Precious Onobraigho, Eugene Omoike, Mehrnaz Hojjati

**Affiliations:** 1 Internal Medicine, Loma Linda University School of Medicine, Loma Linda, USA; 2 Medicine, University of Missouri School of Medicine, Columbia, USA; 3 Internal Medicine, University of Benin College of Medicine, Benin City, NGA; 4 Internal Medicine, University of Benin College of Medicine, Benin city, NGA; 5 Rheumatology, Loma Linda University School of Medicine, Loma Linda, USA

**Keywords:** national population-based study, mortality, infections, emergency department visit rates, giant cell arteritis (gca)

## Abstract

Background

There is scarcity of national level data on the reasons for Emergency Department (ED) presentation among patients with Giant cell arteritis (GCA) in the United States. This study aims to outline the most common reasons for ED presentation among these patients, and the baseline characteristics and outcomes of ED visits principally for GCA.

Materials and methods

We obtained data from the Nationwide Emergency Department Sample (NEDS) 2018 database. Each ED visit in the NEDS has a principal diagnosis (the main reason for the visit) and can have up to 34 other secondary diagnoses. We searched for ED visits for patients aged ≥50 with any diagnosis of GCA using ICD-10 codes. The most common principal discharge diagnoses were divided into organ systems, and specific principal discharge diagnoses were recorded for ED visits among patients with GCA in descending order of frequency. We then outlined baseline characteristics and outcomes of ED visits with a principal diagnosis of GCA.

Results

There were 20,886 ED visits for patients with GCA in 2018. Infections, as well as rheumatologic and cardiovascular disease were the most common reasons for ED presentation, and GCA was the most common specific principal discharge diagnosis for ED visits. There were 3888 ED visits with a principal diagnosis of GCA. These patients were predominantly elderly females, admitted, Medicare insured, with minimal comorbidity burden, and presented to metropolitan teaching hospitals in the south.

Conclusion

GCA patients are most likely to present to the ED due to their underlying GCA. Infections and CV are also common reasons for presentation to the ED.

## Introduction

Giant cell arteritis (GCA) is a primary systemic vasculitis affecting large and medium arteries. The disease often clinically presents as new-onset headaches, scalp tenderness, jaw claudication, and transient visual impairment. However, constitutional symptoms such as fever, weight loss, night sweats, malaise, proximal myalgias, and muscle stiffness with polymyalgia rheumatic (PMR) are also commonly seen [1]. Despite being the most common vasculitis in adults older than 50, the initial diagnosis of GCA remains clinically challenging. It can be delayed due to the non-specific nature of early presenting symptoms [2,3]. Nevertheless, prompt identification and treatment are crucial to preventing the disease’s most severe or even life-threatening complications, such as permanent vision loss, stroke, and aortic aneurysm/dissection [1]. 

Due to disease and treatment-related comorbidities, GCA is associated with a substantial economic burden and healthcare resource utilization [4,5]. Despite this, extensive national data are needed to describe the most common reasons for Emergency Department (ED) presentation among patients with GCA. In this study, we aimed to use a large national population database to identify the most common reasons for ED presentation among patients with GCA. We also aimed to describe baseline socio-demographic characteristics and outcomes of patients who presented to the ED principally for GCA.

The work was previously presented at the American College of Rheumatology Convergence 2022 conference [6].

## Materials and methods

Data source

We obtained data from the Nationwide Emergency Department Sample (NEDS) 2018 database. The NEDS is the largest publicly available ED database in the United States (US). It was created and maintained by the Agency for Healthcare Research and Quality and is part of the healthcare cost and utilization project (HCUP) databases. The NEDS is a nationally representative stratified sample of 20% US hospital-owned EDs [7]. The 2018 NEDS contains about 35.8 million unweighted ED visits, which is roughly estimated to be about 143 million ED visits. Each ED visit in the NEDS has a principal diagnosis (the main reason for the visit) and can have up to 34 secondary diagnoses (any other diagnosis other than the principal diagnosis).

Inclusion criteria

We searched for ED visits for adult patients aged ≥50 with a principal or secondary diagnosis of GCA using the International Classification of Diseases (ICD)-10 code "M315" & "M316" for the GCA population. This includes patients with new diagnoses of GCA or GCA flare. We included all ED visits for adult patients aged ≥50 without any diagnosis of GCA as the control population.

Statistical analysis

STATA, version 16, was used for analysis. By using a "rank" command in STATA, the most common principal discharge diagnoses (or reason for the ED visit) were divided into categories based on organ system, and the most common specific principal discharge diagnoses were recorded for ED visits among patients with GCA in descending order of frequency. We then outlined baseline socio-demographic characteristics and ED visit outcomes of patients who presented to the ED with a principal diagnosis of GCA.

IRB approval

Since NEDS contains de-identified patient-level data, Institutional Board Review (IRB) review was not sought.

## Results

**Figure 1:**
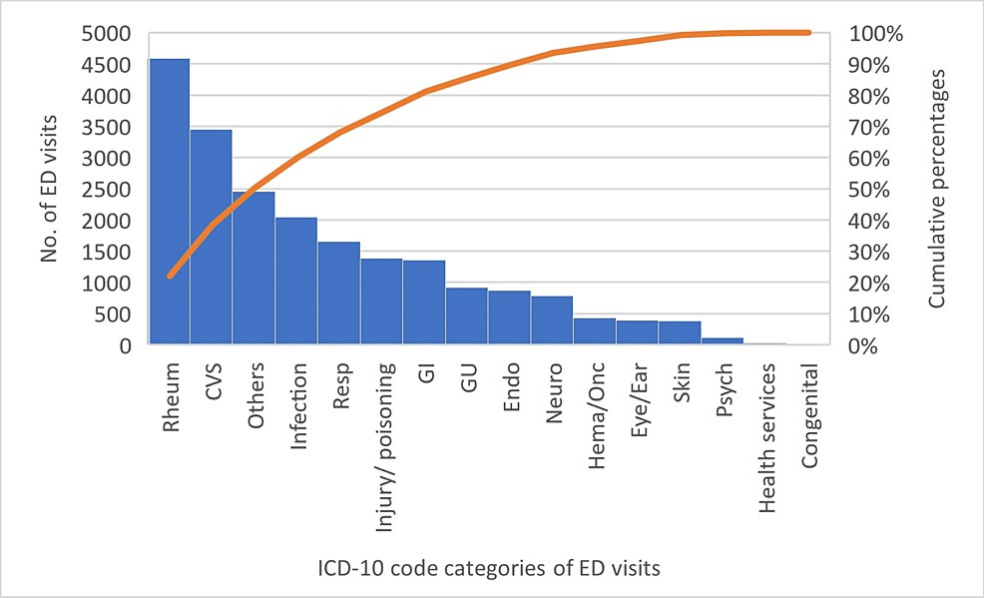
Pareto chart of ICD-10 code categories in descending order of frequency for Emergency Department visits of patients with Giant Cell Arteritis. A line on the secondary axis plots the cumulative percentages of each ICD-10 code category.

Out of 143 million ED visits in 2018, 20,886 (0.015%) were for patients with GCA. Rheumatologic, cardiovascular (CV), infections, and respiratory were the most common reasons for presentation to the ED by organ system category for patients with GCA. GCA, headaches, infectious etiologies such as sepsis, pneumonia, urinary tract infections, and hypertensive heart disease with chronic kidney disease and heart failure were the most common specific principal discharge diagnosis of GCA patients who presented to the ED. See Table 1 and Figure 1.

**Table 1:** ICD-10 code categories and specific principal discharge diagnosis for Emergency Department visits of patients with Giant Cell Arteritis * in 2018. * Emergency department visits for patients aged ≥50 years with any (i.e either principal or secondary) diagnosis of Giant Cell Arteritis. COPD: Chronic obstructive pulmonary disease. Organ system categories with values of 10 and below were excluded from the table to prevent any potential patient identification ** This number included admissions with principal ICD 10 code of "M316" only

ICD 10 Code Category by organ system	Number of ED visits (%)
Certain infections and parasitic diseases	2047 (9.8)
Neoplasms & diseases of the blood and blood-forming organs and certain disorders involving the immune mechanism	432 (2.1)
Endocrine, nutritional, and metabolic diseases	871 (4.2)
Mental, behavioral, and neurodevelopmental disorders	112 (0.5)
Diseases of the nervous system	781 (3.7)
Diseases of the eye and adnexa and Ear	395 (1.9)
Diseases of the circulatory system	3448 (16.5)
Diseases of the respiratory system	1655 (7.9)
Diseases of the digestive system	1359 (6.5)
Diseases of the skin and subcutaneous tissue	387 (1.9)
Diseases of the musculoskeletal system and connective tissue	4590 (22)
Diseases of the genitourinary system	920 (4.4)
Congenital malformations, deformations, and chromosomal abnormalities	15 (0.1)
Symptoms, signs, and abnormal clinical laboratory findings not elsewhere classified	2458 (11.8)
Injury, poisoning, and certain other consequences of external causes	1387 (6.6)
Factors influencing health status and contact with health services	31 (0.1)
Specific principal discharge diagnosis	Number of ED visits (%)
Giant cell arteritis	3659 (17.5) **
Sepsis	1274 (6.1)
Headache	554 (2.7)
Pneumonia	373 (1.8)
Hypertensive heart and CKD with heart failure	363 (1.7)
Urinary tract infection	314 (1.5)
Cerebral infarction	287 (1.4)
Acute kidney failure	279 (1.3)
Chest pain	268 (1.3)
Acute COPD exacerbation	255 (1.2)

Among GCA patients who presented to the ED, 3,888 of these visits were principal because of GCA. ED visits principally because of GCA were predominantly elderly (mean age of 73 years), females (67.7%), admitted (59.2%), Medicare insured (77.2), Charlson comorbidity index score of less than 3 (78.4%) and presented to metropolitan teaching hospitals in the south. See Table 2.

**Table 2:** Baseline characteristics of patients who presented to the Emergency Department principally due to Giant Cell Arteritis compared to the control population in the United States in 2018. Abbreviations: * Emergency department visits for patients aged ≥50 years with Giant Cell Arteritis as the “principal” diagnosis using ICD 10 codes "M315" and "M316", ** Control population:  ED visits for patients aged ≥50 years without any diagnosis of GCA, LOS: Length of inpatient stay for those admitted, Charge: charge for Emergency department visit, USD: United States dollars, Adults: 18 years and above, pediatrics: less than 18 years, ***Income: Median household income national quartile for patient’s Zip code, SNF: Skilled nursing facility, COPD: Chronic obstructive pulmonary disease.

Variables	Principal diagnosis of GCA* (n=3,888)	Control population** (54.4 million)	p-value
Mean Age, years	73	67.4	<0.0001
Female, %	67.7	54.5	<0.0001
Mean LOS, days	4.1	5.1	<0.0001
Aggregate LOS, days	9543	72.7 million	
Mean charge, USD	4822	5234	0.121
Aggregate charge, USD	14.9 million	238 billion	
Charlson comorbidity index score, %			0.0124
0-2	78.4	81.8	
≥3	21.6	18.2	
Income***, quartile, %			0.0002
0-25	26.4	33.2	
26-50	26.7	26.9	
51-75	24.1	21.1	
76-100	22.8	18.8	
Insurance, %			<0.0001
Medicare	77.2	58.9	
Medicaid	6.9	12.9	
Private	13.5	22.6	
Self-pay	2.5	5.6	
Hospital location			0.0003
Rural hospital, %	10.8	16.5	
Metropolitan Hospital, %	89.2	83.5	
Hospital teaching status, %			0.0001
Non-teaching	32.2	40.5	
Teaching	67.8	59.5	
Weekend visit, %	24.4	26.9	0.0932
Region of hospital, %			0.0007
Northeast	25.7	19.3	
Midwest	23.8	22.6	
South	32.2	38.5	
West	18.3	19.6	
Disposition, %			0.2738
Routine	37.8	67.2	
Transfer to a short-term hospital	1.2	2.5	
Transfer to other facilities (including SNF, ICF, and others)	0.4	2.1	
Home Health Care	0.6	0.5	
Leave Against Medical Advice	0.9	1.3	
Admitted	59.2	26.1	

## Discussion

As the US population ages, the incidence and prevalence of GCA are projected to increase over the next several decades [8]. Healthcare resource utilization and direct medical cost due to GCA are likewise expected to increase because of rising disease burden. By 2050, it is estimated that the US alone will have spent $1.13 billion on inpatient GCA management and over $76 billion on managing GCA-associated visual impairment [9]. Thus, understanding the factors contributing to ED presentation and subsequent hospitalization in patients with GCA may allow us to develop strategies to prevent ED presentation and reduce healthcare costs and utilization of these patients. In the present study, we analyzed patients with a diagnosis of GCA who presented to the ED using the largest ED US national database. To the best of our knowledge, this is the first study to look at national estimates of ED utilization by patients with GCA in the US. The results of this study provide critical epidemiological findings regarding the extent of disease burden and healthcare utilization. Our findings indicate that patients with GCA are most likely to present to the ED because of their underlying GCA, emphasizing the severe morbidity associated with GCA.

We found that the mean age for patients presenting to the ED with a primary diagnosis of GCA was 73 years old, which was significantly higher than the control population (73 years vs. 67.4 years; p <0.0001), and consistent with previous reports that the mean age of GCA onset to be 72 years [10]. However, our findings are lower than recent reports that have suggested a rise in the age of onset between 75 to 79.2 years over the last two decades [11-13]. While epidemiologic estimates may vary between regions, our findings indicate that GCA patients are older than the age-matched adult population aged ≥50 years presenting to the ED. The finding that GCA patients more often present to metropolitan (89.2%), and teaching (67.8%) hospitals than the general population may further allude to the greater diagnostic capabilities of academic institutions, especially for the ability to perform diagnostic imaging and temporal arterial biopsy.

Musculoskeletal and connective tissue-related diseases were our study's most common principal discharge diagnosis organ system category (22% of cases). This is expected given that PMR is included within this organ system category and is concurrently seen in 40-60% of GCA cases. These findings may also be related to other comorbid conditions, including osteoporosis-related fractures because of long-term steroid use. Previous studies have reported significantly higher rates of osteoporosis and related fractures among patients with GCA compared to age-matched controls [14-16]. One study found that approximately 1 in 4 patients with GCA will experience at least 1 osteoporosis-related fracture in their lifetime [15]. It may thus be important for clinicians to consider more frequent bone density testing to evaluate and treat osteoporosis in patients with GCA, especially when on steroids.

The relatively high incidence of sepsis (6.1%) and infections (9.8%) in our study may also be a result of long-term steroid use for GCA or the use of other immunosuppressants in GCA patients [17]. In agreement with our findings, several studies have demonstrated that patients with GCA are at increased risk for systemic infections, especially within the first year after diagnosis [18,19]. The higher rate of infections associated with steroid use emphasizes the need for increased use of alternative steroid-sparing treatments, including newer treatments such as Tocilizumab, the only approved biologic for GCA [17].

The finding that CV disease was the second most common discharge diagnosis category (16.5%) reflects the increased risk for CV comorbidities in GCA. This agrees with prior cohort and meta-analysis studies showing that GCA is associated with a substantially increased risk for myocardial infarction, stroke, thoracic aortic aneurysms and dissections, and peripheral vascular disease [15,20,21]. The high rate of morbidity and mortality associated with these conditions highlights the importance of screening and managing CV risk factors in GCA patients. Although using low-dose aspirin and statin as adjunctive treatment in GCA has not consistently shown mortality benefits in prior studies, strategies to manage CV risk factors are needed for optimal outcomes for GCA patients [22-25].

Our study has several strengths. Using a national database allowed us to analyze a large sample size of GCA ED visits representative of the entire US population. Since principal diagnosis is the main reason for ED visits in the NEDS, we were able to study ED visits principally for GCA and compare this to the general age-matched population of non-GCA patients. Our study provided useful insights into baseline socio-demographic characteristics for patients who presented to the ED principally due to GCA.

Our study has limitations. NEDS is a claims database based on ICD coding; hence, there may be errors due to coding. Our data cannot report the percentage of patients that met the American College of Rheumatology (ACR) classification criteria for GCA or had temporal artery biopsy-proven disease. This study is subject to the bias and limitations of a retrospective study. The database provides data on ED visits rather than individual patients; therefore, patients who presented to the ED on multiple occasions will be counted multiple times for each ED visit [26,27]. We cannot differentiate between ED visits for patients with newer diagnoses and those for a flare of previously diagnosed GCA. NEDS 2018 dataset does not contain data on race, medication (such as steroids, other immunosuppressants, and new biologics like Tocilizumab) use, laboratory values (such as inflammatory markers), and imaging findings (such as aortitis on chest imaging or temporal artery ultrasound).

## Conclusions

GCA patients are most likely to present to the ED due to their underlying GCA. Infections, CV, and rheumatologic diseases are common reasons for presentation to the ED. GCA patients who presented to the ED principally due to GCA are most likely to be elderly females and admitted. Management of GCA and medical comorbidities (such as CV), measures for prevention, and early detection of infections while on immunosuppressants in the outpatient setting are essential in preventing ED visits of GCA patients.
